# Home care quality indicators based on the Resident Assessment Instrument-Home Care (RAI-HC): a systematic review

**DOI:** 10.1186/s12913-020-05238-x

**Published:** 2020-04-29

**Authors:** Aylin Wagner, René Schaffert, Nathalie Möckli, Franziska Zúñiga, Julia Dratva

**Affiliations:** 1 ZHAW Zurich University of Applied Sciences, School of Health Professions, Institute of Health Sciences, Technikumstrasse 71, 8401 Winterthur, Switzerland; 2 University of Basel, Medical Faculty, Department of Public Health, Institute of Nursing Science, Bernoullistrasse 28, 4056 Basel, Switzerland; 3 University of Basel, Medical Faculty, Klingelbergstrasse 61, 4056 Basel, Switzerland

**Keywords:** Systematic review, Quality indicators, Home care, Home-based care, Nursing, InterRAI, Validity, Reliability

## Abstract

**Background:**

One way of measuring the quality of home care are quality indicators (QIs) derived from data collected with the Resident Assessment Instrument-Home Care (RAI-HC). In order to produce meaningful results for quality improvement and quality comparisons across home care organizations (HCOs) and over time, RAI-HC QIs must be valid and reliable. The aim of this systematic review was to identify currently existing RAI-HC QIs and to summarize the scientific knowledge on the validity and reliability of these QIs.

**Methods:**

A systematic review was performed using the electronic databases PubMed, CINAHL, Embase, PsycINFO and Cochrane Library. Studies describing the development process or the psychometric characteristics of RAI-HC QIs were eligible. The data extraction involved a general description of the included studies as well as the identified RAI-HC QIs and information on validity and reliability. The methodological quality of the identified RAI-HC QI sets was assessed using the Appraisal of Indicators through Research and Evaluation (AIRE) instrument.

**Results:**

Four studies out of 659 initial hits met the inclusion criteria. The included studies described the development and validation process of three RAI-HC QI sets comprising 48 unique RAI-HC QIs, which predominantly refer to outcome of care. Overall, the validity and reliability of the identified RAI-HC QIs were not sufficiently tested. The methodological quality of the three identified RAI-HC QI sets varied across the four AIRE instrument domains. None of the QI sets reached high methodological quality, defined as scores of 50% and higher in all four AIRE instrument domains.

**Conclusions:**

This is the first review that systematically summarized and appraised the available scientific evidence on the validity and reliability of RAI-HC QIs. It identified insufficient reporting of RAI-HC QIs validation processes and reliability as well as missing state-of-the-art methodologies. The review provides guidance as to what additional validity and reliability testing are needed to strengthen the scientific soundness of RAI-HC QIs. Considering that RAI-HC QIs are already implemented and used to measure and compare quality of home care, further investigations on RAI-HC QIs reliability and validity is recommended.

## Background

The change of populations’ age-structure has a significant impact on health systems worldwide and, in particular, poses challenges for home care [1]. Home care in the context of this study is defined as medical and personal care provided by professional nursing staff within clients’ own homes. Globally, the number of people aged 60 and older is expected to double by 2050 [2]. As larger demographic cohorts enter old age and life expectancy increases, more people will live with chronic illnesses, multi-morbidity, as well as functional and cognitive impairments [3]. Findings have shown that the large majority of older people in need of care prefer to remain in their known physical and social environment for as long as possible, leading to increased demand for home care [4]. In order to satisfy peoples’ preferences and to reduce costs of long-term institutional care, many countries have promoted home care in recent decades by shifting resources accordingly [1]. Given the growing importance of home care, it is essential to assess and monitor the quality of the delivered care.

Quality indicators (QIs) are increasingly used to measure, monitor and evaluate health care quality by assessing particular structures, processes, or outcomes. QIs can point to areas where the quality of care is suboptimal, subsequently allowing priorities to be set for quality improvement [5]. Moreover, QIs are used for comparisons of health care quality and thus enable national or international benchmarking [6]. Similar to other measuring instrument, QIs should meet certain quality criteria such as relevance and feasibility and be evaluated for their scientific strength, i.e. their validity and reliability [7]. QIs that do not meet these quality criteria can result in inadequate and misleading information.

In many countries in North America, Europe and Asia-Pacific, quality of home care is assessed with QIs based on the Resident Assessment Instrument-Home Care (RAI-HC or interRAI-HC). RAI-HC was developed in 1994 by the multinational research consortium interRAI. The instrument is a standardized assessment tool to assess long-stay home care clients’ health status, need for care, and basic background information on housing and informal caregivers. The instrument RAI-HC [8–13] and its clinically based scales (e.g. Cognitive Performance Scale, Depression Rating Scale) [14–20] have been validated in several international studies. Although the principal intended use of RAI-HC is to plan care provision, RAI-HC items and scales are also used to derive process and outcome QIs [21]. These RAI-HC QIs are rate-based indicators, i.e. defined by a numerator and denominator, and measure processes or outcomes expected to occur with a certain frequency [22].

To date, no systematic review has been undertaken to summarize the scientific soundness, such as the validity and reliability of RAI-HC QIs, despite the fact that these indicators are implemented in several countries and applied by researchers to measure and compare the quality of home care [23–26]. The only previous overview and quality assessment of QIs for community care by Joling et al. [27] included RAI-HC QIs only partially, as it focused on QIs specifically developed for older people or applied in an older aged sample (i.e. 65 years or older). Therefore, this systematic review aimed to (i) identify all current existing RAI-HC QIs and to (ii) summarize the scientific evidence of RAI-HC QIs validity and reliability.

## Methods

The systematic review was conducted in compliance with The Cochrane Handbook for Systematic Reviews of Interventions [28]. The protocol for the systematic review has been published on PROSPERO (2018: CRD42018110948) and is available at https://www.crd.york.ac.uk/prospero/display_record.php?RecordID=110948.

### Search strategy

The search was carried out using five electronic databases: PubMed, CINAHL, Embase, PsycINFO and Cochrane Library on June 26, 2018. An update of the search was conducted on August 20, 2019, resulting in no additional eligible articles. The search strategy involved both keywords and the Medical Subject Headings (MeSH) combined with the appropriate Boolean connectors (see Additional file 1). In addition, the reference lists of the eligible studies were manually searched for additional relevant articles that had not been identified in the electronic database. Furthermore, we searched for grey literature on websites of relevant organizations (e.g. www.interrai.org) and contacted the study authors of two included articles, namely Burla et al. [29] and Morris et al. [30], to obtain additional information on the QIs definitions used in their study.

### Inclusion and exclusion criteria

Studies were included if they fulfilled the following criteria: (i) the study was conducted in the home care setting, (ii) included adults aged 18 years and older, and (iii) described the development process of RAI-HC QIs or evaluated the psychometric characteristics of RAI-HC QIs.

Studies were excluded if they (i) used RAI-HC or its scales without explicitly using the RAI-HC QIs, (ii) applied already developed RAI-HC QIs for quality measure, (iii) validated the RAI-HC or its scales but not RAI-HC QIs, and (iv) focused on specialized home care for mental health or palliative care. We excluded studies focusing on mental health and palliative care because the needs of these home care clients are different from those of general long-stay home care clients. Therefore, specialized assessment instruments such as the interRAI Palliative Care (PC) and the interRAI Community Mental Health (CMH) are available to assess the needs of mental health and palliative care clients and to plan their care provision. Only recently, QIs have been developed to measure the quality in these contexts [31, 32].

### Screening

The studies identified by the electronic search were entered into the reference management software Endnote X8, and duplicates were removed. The articles were independently screened by two authors (AW and FZ) according to the inclusion criteria first by title and abstract. Non-eligible studies were removed at this stage. Publications included after the title/abstract screening underwent concurrent full-text screening by two reviewers (AW and FZ) for definitive inclusion. Any disagreements that arose between the reviewers were resolved through discussion until consensus was reached.

### Data extraction

Two data extraction forms were developed. First, a structured form was used to describe the included studies with respect to relevant information regarding RAI-HC QIs. The following data were extracted: first author, year of publication, country, study aim, study population, sample size, name of QI set, the number of QIs in the set and a short description of the development and validation process of the QI set. Second, a structured form was used to extract and summarize the identified RAI-HC QIs. For each QI, the following data were extracted: QI description such as name, type (prevalence or incidence), name of the corresponding QI set, and data regarding validity and reliability. Furthermore, the QIs were classified by the study authors (AW and FZ) according to their measure level (outcome or process). Missing data regarding QI definitions were requested from study authors.

### Methodological assessment

We used the Appraisal of Indicators through Research and Evaluation (AIRE) instrument for the methodological assessment of the RAI-HC QIs identified in the articles [33]. AIRE is a validated instrument for a critical appraisal of QIs and has been used in previous scientific publications on QIs [27, 34–36]. The AIRE instrument comprises 20 items, subdivided into four domains: 1. Purpose, relevance and organizational context, 2. Stakeholder involvement, 3. Scientific evidence, and 4. Additional evidence, formulation and usage. Two authors (AW and NM) independently appraised all included QI sets with the AIRE instrument. Information from the included articles as well as QI definitions received from study authors on request were used for the assessment. Standardized scores per domain were calculated. Scores range between 0 and 100%, with a higher score indicating a higher methodological level. A high methodological quality is defined by a score of 50% or higher, which correlates with an overall “agree” or “strongly agree” for each domain.

## Results

### Search results

**Fig. 1:**
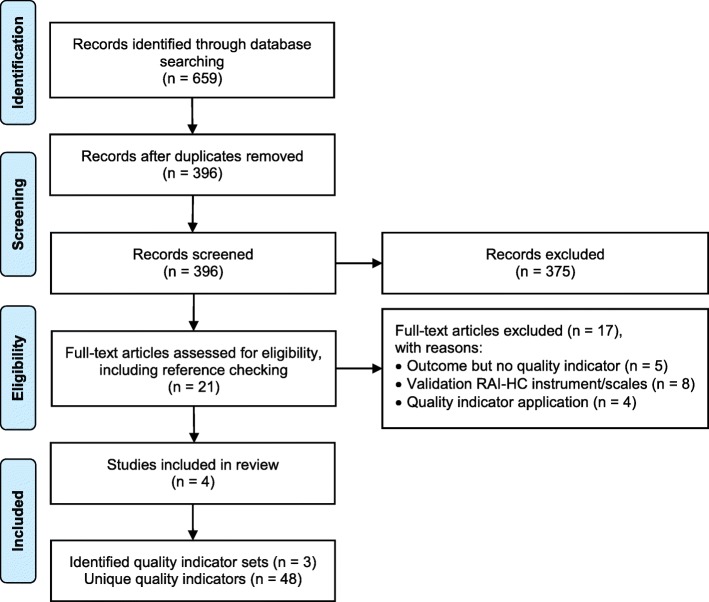
PRISMA flow diagram of study selection

The systematic review identified 659 potentially relevant studies. The PRISMA flow diagram for the study selection process and reasons for exclusion are shown in Fig. 1. After removal of duplicates and title/abstract screening, 21 studies were potentially relevant. Four studies met the selection criteria after full-text screening [29, 30, 37, 38]. Reference tracking of the eligible studies identified no additional article. The four included studies describe three RAI-HC QI sets comprising 48 unique RAI-HC QIs.

### Description of studies

Table 1 shows the main characteristics of the selected papers. Three studies specified the development and validation process of separate RAI-HC QI sets, namely, interRAI’s 1st generation QI set developed by Hirdes et al. [37], the Swiss RAI-HC QI set developed by Burla et al. [29], and interRAI’s 2nd generation QI set developed by Morris et al. [30]. One study by Dalby et al. [38] explored the effects of risk adjustment for interRAI’s 1st generation QI set. The included studies were not primarily aimed at examining the validity and reliability of RAI-HC QIs, and only partially reported such results.

**Table 1:** Included articles in review

1st author, Year of publication	Country	Study aim	Study population (sample size)	Name of QI set	Number of QIs	Development and validation process of QI set
Hirdes, 2004 [37]	Canada and USA	To develop home care QIs based on RAI-HC.	Persons receiving home care services for at least 30 days (*n* = 14,293 clients)	interRAI’s 1st generation QI set	Total: 22 Outcome: 19 Process: 3	Step 1: Identification of candidate QIs based on literature review, focus groups and expert meetings. Step 2: Ranking of QIs by investigators. Step 3: Empirical testing of QIs (denominator size, relative frequency and variation) and development of risk adjustment based on cross-national data.
Dalby, 2005 [38]	Canada	To explore the effect of risk adjustment for interRAI’s 1st generation QI set.	Persons aged 18 years and older receiving home care services (*n* = 22 HCOs)	interRAI’s 1st generation QI set	Total: 22 Outcome: 19 Process: 3	No QI development, but investigation of three approaches of risk adjustment and comparison of unadjusted and risk-adjusted QI rates.
Burla, 2010 [29]	Switzerland	To develop and examine home care QIs based on RAI-HC for Switzerland.	Persons aged 18 years and older receiving home care services (*n* = 1808 clients, 45 HCOs)	Swiss RAI-HC QI set	Total: 29 Outcome: 27 Process: 2	Step 1: Identification of candidate QIs based on interRAI’s 1st generation QI set and development of new QIs for the Swiss context. Step 2: Rating of QIs in focus groups with health care professionals (nominal group technique). Step 3: Empirical testing of QIs (denominator size, relative frequency and variation) based on Swiss home care data. Step 4: Interrater reliability testing of subset of QIs.
Morris, 2013 [30]	Europe, Canada, USA	To develop 2nd generation home care QIs based on RAI-HC.	Persons aged 65 years and older receiving home care services (*n* = 335,544 clients, 1654 HCOs)	interRAI’s 2nd generation QI set	Total: 23 Outcome: 22 Process: 1	Step 1: Identification of candidate QIs based on interRAI’s 1st generation QI set and QIs from other settings. Step 2: Evaluation of QIs by representatives of HCOs in one-on-one discussions and focus groups. Review of QIs by interRAI’s cross-national program development committee. Step 3: Empirical testing of QIs (denominator size, relative frequency, variation, factor analysis) and development of risk adjustment based on cross-national data.

*Abbreviations*: *QI* Quality indicator, *RAI-HC* Resident Assessment Instrument-Home Care, *HCO* Home care organization

### Development and validation process of quality indicator sets

#### InterRAI’s 1st generation quality indicator set

Hirdes et al. [37] described the development and validation process of interRAI’s 1st generation QI set. Initially, a literature review was conducted to identify candidate QIs used in other care settings. In addition, expert meetings and focus groups with health professionals and older adults were conducted to identify further candidate QIs. The literature review (e.g. search string, results, synthesis) and process of the focus groups were not described in the article. In total, 74 candidate QIs were generated which were first prioritized by the investigators according to their relevance to different types of home care clients and second, ranked in terms of their appropriateness for the different types of home care clients. The average ranks were then used to reduce the number of candidate QIs. The article offers no information on the ranking process, the average ranks and the criteria determining when a QI was considered inappropriate. The QIs were empirically tested with regard to relative frequencies, variation and denominator size based on data from 14,293 home care clients from Ontario and Michigan. QIs with a relative frequency of less than 5% and more than 95%, respectively, and too little variation (interquartile range) among HCOs were excluded. The article provides no information on the basis of which criteria QI variation was considered as insufficient. Based on the overall study results, a final set of 22 QIs was defined, for which risk adjustment methods such as client-level covariates and an agency-level adjuster, namely, the Agency Intake Profile (AIP), has been quantitatively evaluated [37].

Dalby et al. [38] further explored the effects of risk adjustment for interRAI’s 1st generation QI set. Based on data of 22 HCOs in Ontario and the Winnipeg Regional Health Authority (WRHA) in Manitoba, three types of risk adjustment methods were applied, namely, client covariates only, client covariates plus AIP, and client covariates plus the intake Case Mix Index associated with the Resource Utilization Groups version III for Home Care methodology [39]. Based on the three approaches, risk adjustment showed substantial effects on the organization level but only small effects on the regional level. On the regional level, the risk adjustment process minimized the differences in QI rates between Ontario and the WRHA compared with the unadjusted rates. On the organization level, risk adjustment had an impact on agency rankings across the set of QIs. While the HCOs in Ontario benefited from the risk adjustment, i.e. they were less likely to be ranked among the worst performers, the opposite was true for the HCOs in the WRHA [38].

#### The Swiss RAI-HC quality indicator set

The development and validation process of the Swiss RAI-HC QI set was described by Burla et al. [29]. Based on interRAI’s 1st generation QI set and by creating new QIs for the Swiss context with support of various experts, 29 candidate QIs were chosen. The QIs were rated according to their appropriateness of measuring home care quality in focus groups with health care professionals from HCOs using the nominal group technique (NGT). The rating process and the QI ratings were presented in the paper. Relative frequencies of all candidate QIs and the variation of 24 QIs (due to small sample size) were examined based on data from 1808 home care clients from Switzerland. QIs with a relative frequency of less than 5% or more than 95% and/or a low variation (interquartile difference less than 6%) were specified as inadequate. Furthermore, due to small sample size interrater reliability was analyzed in only 18 QIs. For this purpose, 24 home care clients were independently assessed by two assessors. The results of the expert rating, frequencies, variation, and interrater reliability were summarized and only QIs that met at least three of the four criteria were defined as appropriate, resulting in a final core-set of 19 QIs [29].

#### InterRAI’s 2nd generation quality indicator set

Morris et al. [30] described the development and validation process of interRAI’s 2nd generation QI set. A list of 64 candidate QIs was compiled including both 1st generation QIs as well as newly designed QIs, drawn from lists of QIs that interRAI has considered for home care, post-acute care, and long-term care. The QIs were empirically tested with regard to relative frequencies and variation based on a sample of 335,544 home care clients from the U.S., Canada and Europe. QIs with a relative frequency of less than 3% were excluded. Variation was examined by comparing scores of top performing HCOs (5th percentile) with scores of the poorest performing HCOs (95th percentile). QIs with less than a two-fold difference in scores from the 5th to 95th percentile, thus not discriminatory enough, were dropped. Additionally, factor analysis was conducted for eight functional QIs measuring improvement and decline in cognition, communication, activities of daily living (ADL) and instrumental activities of daily living (IADL) to confirm that functional decline and improvement QIs say something different about the performance of HCOs. The QIs were further evaluated by representatives of HCOs in focus groups and one-on-one discussions to determine if the QIs could be influenced by efforts of HCOs. The remaining QIs were reviewed regarding face validity by 16 members of interRAI’s cross-national program development committee. Each QI had to be approved by at least 70% of the members to remain on the QI list. Rating results, rating criteria and the exact method of consensus are not indicated in the article. The definite QI set comprised 23 QIs. For the QI set, a new approach of risk adjustment was employed with more complex covariate structures, a longer list of covariates, and direct stratification [30].

### Validity and reliability of RAI-HC quality indicators

Table 2 gives an overview of the characteristics of the 48 identified RAI-HC QIs and summarizes findings on their validity and reliability. Face validity was examined for all identified QIs based on expert opinion. However, only Burla et al. [29] described rating results for 29 Swiss RAI-HC QIs, with seven QIs rated as inappropriate to measure quality of home care. Reliability was addressed explicitly by Burla et al. [29] providing results of interrater reliability testing for the RAI-HC items used for the calculation of 18 Swiss RAI-HC QIs. Interrater reliability was calculated by Burla et al. [29] using Kappa and Yules. The higher Kappa/Yules (range 0–1), the higher the agreement between the two independent assessors. Twelve QIs showed moderate (Kappa/Yules values 0.40–0.59) or good interrater reliability (Kappa/Yules values ≥0.60) and six QIs had insufficient interrater reliability (Kappa/Yules values < 0.40) [29, 40].

**Table 2:** Characteristics of identified RAI-HC quality indicators

QI	Measure level^**a**^	Type^**b**^	QI set affiliation^**c**^			Face validity^**d**^			Interrater reliability^**e**^
			interRAI 1st	Swiss RAI-HC	interRAI 2nd	interRAI 1st	Swiss RAI-HC	interRAI 2nd	Swiss RAI-HC
ADL	O	I	✓	✓		C	A		≥ 0.60
ADL decline	O	I			✓			B	
ADL improvement	O	I			✓			B	
IADL	O	I		✓			A		≥ 0.60
IADL decline	O	I			✓			B	
IADL improvement	O	I			✓			B	
Rehabilitation potential and no therapies	P	P	✓	(✓)		C	A		N/A
Decline independency	O	P		✓			A		N/A
Difficulties in communication	O	I	✓	✓		C	A		N/A
Communication decline	O	I			✓			B	
Communication improvement	O	I			✓			B	
Bladder incontinence	O	I	✓	✓		C	D		≥ 0.60
Bladder decline	O	I			✓			B	
Bladder improvement	O	I			✓			B	
Bowel incontinence	O	I		(✓)			D		≥ 0.60
Obstipation	O	I		(✓)			A		≥ 0.60
Skin ulcer	O	I	✓	✓		C	A		< 0.40
Mouth problems	O	P		✓			A		0.40–0.59
Cognitive function	O	I	✓	✓		C	D		0.40–0.59
Cognitive decline	O	I			✓			B	
Cognitive improvement	O	I			✓			B	
Delirium	O	P	✓	(✓)		C	A		N/A
Negative mood	O	P	✓	✓		C	A		< 0.40
Mood decline	O	I			✓			B	
Mood improvement	O	I			✓			B	
Negative mood without intervention	O	P		(✓)			A		< 0.40
Social isolation with distress	O	P	✓	✓	✓	C	A	B	0.40–0.59
Does not go out but used to	O	P			✓			B	
Continued caregiver distress	O	P		✓	✓		A	B	< 0.40
Falls	O	P	✓	✓	✓	C	D	B	≥ 0.60
Neglect or abuse	O	P	✓	✓		C	A		N/A
Injuries	O	P	✓		✓	C		B	
Hospital, ED, emergent care	O	P	✓		✓	C		B	
Daily severe pain	O	P	✓	✓	✓	C	A	B	0.40–0.59
Inadequate pain control	O	P	✓	✓	✓	C	A	B	< 0.40
Pain improvement	O	I			✓			B	
Dehydration	O	P	✓	✓		C	A		0.40–0.59
Weight loss	O	P	✓	✓	✓	C	A	B	N/A
Weight change (undesired)	O	I		(✓)			D		N/A
Weight change (unfavourable)	O	I		(✓)			D		N/A
Inadequate meals	O	P	✓			C			
Difficulty in locomotion and no assistive device	O	P	✓			C			
Impaired locomotion in home	O	I	✓	✓		C	A		≥ 0.60
No medication review by MD	P	P	✓	✓		C	A		N/A
Inconsistent drug intake	O	P		(✓)			A		N/A
Hearing	O	P		(✓)			A		< 0.40
Eyesight	O	P		(✓)			D		N/A
No flu vaccination	P	P	✓		✓	C		B	

*Abbreviations*: *ADL* Activities of daily living, *ED* Emergency department, *IADL* Instrumental activities of daily living, *MD* Medical doctor, *QI* Quality indicator, *RAI-HC* Resident Assessment Instrument-Home Care

^a^ O = Outcome, P = Process, classified by authors

^b^ I = Incidence-based measure (measures changes in a client’s health status from one time point to another), P = Prevalence-based measure (measures client's health status at a single point in time)

^c^ interRAI 1^st^ = interRAI's 1^st^ generation QI set by Hirdes et al. [37], Swiss RAI-HC = Swiss RAI-HC QI set by Burla et al. [29], interRAI 2^nd^ = interRAI's 2^nd^ generation QI set by Morris et al. [30], ✓ = part of the QI set, (✓) = part of the QI set but not core set (only for Swiss QIs)

^d^ Face validity was assessed for all three QI sets:

A = Face validity assessed with nominal group technique and rated as appropriate

B = Face validity assessed with unstructured consensus process (approved by 70% of experts) and rated as appropriate

C = Rating process of face validity not described but rated as appropriate

D = Face validity assessed with nominal group technique and rated as inappropriate

^e^ Interrater reliability was tested by Burla et al. [29] for 18 Swiss RAI-HC QIs, values indicate kappa/yules values, N/A = not applicable: interrater reliability not tested due to small sample size**.** Kappa/yules values < 0.40 were interpreted as insufficient reliability

### Methodological characteristics of RAI-HC quality indicator sets

The methodological quality of the three identified RAI-HC QI sets varied according to the AIRE instrument domain scores (see Table 3). The AIRE instrument domain ratings ranged from 0 to 69%. None of the QI sets reached high methodological quality, defined as scores of 50% or higher in all four AIRE instrument domains.

**Table 3:** Methodological characteristics of RAI-HC quality indicator sets (AIRE instrument)

	interRAI’s 1st generation QI set [37]	Swiss RAI-HC QI set [29]	interRAI’s 2nd generation QI set [30]
**Domain 1: Purpose, relevance and organizational context**	**60%**	**60%**	**47%**
The purpose of the indicator is described clearly	4	4	3.5
The criteria for selecting the topic of the indicator are described in detail	2.5	4	3
The organizational context of the indicator is described in detail	4	3.5	2
The quality domain the indicator addresses is described in detail	2.5	1.5	2.5
The health-care process covered by the indicator is described and defined in detail	1	1	1
**Domain 2: Stakeholder Involvement**	**44%**	**28%**	**56%**
The group developing the indicator includes individuals from relevant professional groups	3.5	2.5	4
Considering the purpose of the indicator, all relevant stakeholders have been involved at some stage of the development process	2.5	2	3
The indicator has been formally endorsed	1	1	1
**Domain 3: Scientific evidence**	**11%**	**0%**	**0%**
Systematic methods were used to search for scientific evidence	1.5	1	1
The indicator is based on recommendations from an evidence-based guideline	1.5	1	1
The supporting evidence has been critically appraised	1	1	1
**Domain 4: Additional evidence, formulation and usage**	**69%**	**48%**	**54%**
The numerator and denominator are described in detail	4	4	4
The target patient population of the indicator is defined clearly	4	2	2
A strategy for risk adjustment has been considered and described	4	1	4
The indicator measures what it is intended to measure (validity)	2.5	2.5	2.5
The indicator measures accurately and consistently (reliability)	1	3.5	1
The indicator has sufficient discriminative power	3.5	3	3.5
The indicator has been piloted in practice	1	1	1
The efforts needed for data collection have been considered	4	4	4
Specific instructions for presenting and interpreting the indicator results are provided	3.5	1	1.5

*Abbreviations*: *AIRE* Appraisal of Indicators through Research and Evaluation_;_*QI* Quality indicator

*Item scores:* Each item score ranges from 1 to 4 with 1 = strongly disagree (confident that the criterion has not been fulfilled or no information was available), 2 and 3 = disagree/agree (unsure whether the criterion has been fulfilled) and 4 = strongly agree (confident that the criterion has been fulfilled) [33]

*Domain score calculation:* Domain scores for the four AIRE instrument domains were calculated as follows: first, the two authors’ scores per item were summed up and divided by two to obtain an average rating per item; second, the average item scores were summed up per domain to obtain the domain score; and third, the domain score were standardized using the following formula: (total score per domain - minimum possible score) / (maximum possible score - minimum possible score) × 100%

*High methodological quality of QI set:* If score ≥ 50% across all four AIRE instrument domains

InterRAI’s 1st generation QI set [37] and the Swiss RAI-HC QI set [29] scored 50% or higher in the first AIRE instrument domain demonstrating good evidence for “purpose, relevance and organizational context”. InterRAI’s 1st generation QI set [37] and the Swiss RAI-HC QI set [29] scored poorly in the domain “Stakeholder involvement” due to a lack of involvement of relevant stakeholders at some stage of the development process. The three QI sets scored between 0 and 11% in the domain “Scientific evidence”. None of the three studies performed a systematic review to investigate evidence-based guidelines supporting QIs nor did they examine the relationships between care processes and outcomes. The domain “Additional evidence, formulation and usage” indicated for interRAI’s 1st and 2nd generation QI set [30, 37] a good methodological quality with scores of 50% or higher.

## Discussion

In this systematic review, the three identified RAI-HC QI sets contained a total of 48 unique QIs covering various health domains and predominantly referring to outcome of care. To be able to draw valid conclusions from QIs, it is relevant to establish the validity and reliability of the QIs. The methodological assessment of the three QI sets, however, indicated relatively low methodological quality and a lack of evidence of validity and reliability.

QI’s validity, such as face, content, construct, and criterion aspects, should either be supported by scientific literature or be examined. When addressing scientific evidence, it is recommended to follow a systematic approach and to search both for scientific as well as grey literature, and not only to identify articles regarding the validity of QIs but also articles that discuss the outcome of interest [41]. However, this review showed that the above recommendations were not applied in the development processes of extant RAI-HC QIs. The majority of identified RAI-HC QIs were adopted from other care settings or other interRAI QI sets, and in none of the included studies was a systematic review carried out to identify candidate QIs or to address scientific evidence on QIs.

In practice, and in the absence of scientific literature on QIs, face validity of QIs is often assessed based on the opinions and experience of experts [42]. Commonly used structured consensus techniques for QI development combining expert opinion with available evidence are the Delphi techniques [43], the RAND/UCLA Appropriateness Method [44] and the NGT [45]. The advantages of these approaches are that experts can be included anonymously and the undue social influence processes toward expert consensus can be minimized [42]. In all three identified RAI-HC QI sets, experts were involved to evaluate the face validity of QIs, however, such a structured approach was only applied by Burla et al. [29] using the NGT in the development process of the Swiss RAI-HC QIs. Hirdes et al. [37] did not report on the exact process of assessing face validity for interRAI’s 1st generation QIs and the consensus process used by Morris et al. [30] for interRAI’s 2nd generation QIs was incompletely described. Considering that consensus for both interRAI QI sets was reached in an unstructured way and by face-to-face discussion only, rating results could be biased toward the opinions of influential or persuasive experts.

Other forms of validity such as content, construct and criterion validity of RAI-HC QIs have not been examined. Admittedly, similar to nursing home QIs [46, 47], criterion validity of home care QIs can be difficult to measure because few, if any, valid “gold standard” measurements exist that can be used for comparison. While some of the identified RAI-HC QIs are calculated using validated RAI-HC scales (e.g. Cognitive Performance Scale, Depression Rating Scale and Pain Scale) [14, 16–18], the use of validated scales does not necessarily ensure the validity of the corresponding QI. RAI-HC scales have not been developed to evaluate quality of care. In the absence of QI “gold standard” measurements, construct validity of QIs is often examined instead of criterion validity, e.g. by assessing correlations between process and outcome QIs [36, 48, 49]. However, such assessments were not carried out in the reviewed articles since there are only few process RAI-HC QIs.

The review revealed that the reliability of the identified RAI-HC QIs has hardly been tested so far. For example, interrater reliability of RAI-HC QIs, respectively the underlying RAI-HC items, was only assessed by Burla et al. [29] for a subset of Swiss RAI-HC QIs. Burla et al. [29] found moderate or good interrater reliability for 12 QIs and insufficient interrater reliability for six QIs. Possible reasons for poor interrater reliability are difficulty to detect certain health conditions (e.g. delirium), a high rate of true clinical change and the fluctuations of symptoms and function, misinterpretation of assessment instructions, or poorly designed assessment items [50]. Further, Burla et al. [29] point out that the interrater reliability findings of the 18 QIs must be interpreted cautiously due to the small sample size (only 24 home care clients were assessed). In contrast to the RAI-HC QIs, the psychometric properties of the instrument RAI-HC, i.e. its items and scales, has been tested more thoroughly. These validation studies show overall acceptable reliability results [8, 10, 11, 13]. Nevertheless, good reliability of RAI-HC data does not guarantee the reliability of QIs based on these data nor the ability to be applied in quality comparisons over time or between providers [51, 52].

In addition, the reported validation process of RAI-HC QIs in the included articles did not involve precision tests to determine the reliability of QIs for distinguishing real differences in performance. In the reviewed studies, between-provider variation was assessed by comparing QI rates (e.g. interquartile range) among HCOs and geographic regions, whereby for interRAI’s 1st and 2nd generation QIs unadjusted as well as risk-adjusted rates and for the Swiss RAI-HC QIs only unadjusted rates were computed. However, the possibility that a substantial amount of between-provider variation is attributable to random variation has not been considered. Chance can cause substantial differences in performance in the absence of true quality differences [6]. The empirical evaluation of QIs for the acute care [6, 53–55] and nursing home setting [46, 56–59] includes other statistical methods to test for reliability such as the intra-class correlation coefficient or more advanced modeling techniques, such as multilevel or Bayesian-based estimation as well as thorough risk adjustments. To determine whether RAI-HC QIs have the ability to consistently measure quality differences in home care, the application of the above mentioned statistical procedures should be considered.

### Strengths and limitations

To the authors’ knowledge, this is the first attempt to identify and summarize, in a systematic way, the scientific evidence on validity and reliability of RAI-HC QIs, thereby identifying gaps for potential improvement in future validation studies. The review was limited by the small number of articles available. While it cannot be ruled out that validation studies regarding RAI-HC QIs may not have been published in peer-reviewed journals, grey literature searches did not provide additional publications. To the best of our knowledge, we have reviewed all published work on the validity and reliability of RAI-HC QIs. Due to the poor reporting of methodology and results, it is difficult to draw a firm conclusion on the overall validity and reliability of the QIs. Furthermore, the QI assessment with the AIRE instrument was hindered by the limited information in the validation processes of the RAI-HC QI sets. Thus, the AIRE instrument rating results have to be interpreted cautiously.

## Conclusion

Based on the description of the RAI-HC QI sets, the validation processes, and the methodological assessment with the AIRE instrument, this review indicates that the quality of the evidence underpinning the identified RAI-HC QIs is weak and information about validity and reliability is scarce. QIs that are not valid and reliable result in an inaccurate or unreliable measure of the quality of care and, therefore, are neither useful for identifying poor nor desirable quality of care [7]. In addition, information on the methodological quality of QIs is crucial for different stakeholders such as health care providers or policy-makers when selecting QIs for their intended use. This review provides suggestions as to what additional testing of QIs are needed to strengthen their scientific soundness. Considering that RAI-HC QIs are already implemented and frequently used by HCOs for quality improvement processes but also in scientific research to measure and compare home care quality among HCOs or regions, more evidence of the validity and reliability of RAI-HC QIs is essential.

## Supplementary information

**Additional file 1.** Search strategy used in PubMed.

## Data Availability

Data sharing is not applicable to this article as no datasets were generated or analysed during the current study.

## Abbreviations

ADL: Activities of daily living
AIP: Agency Intake Profile
AIRE: Appraisal of Indicators through Research and Evaluation
ED: Emergency department
HCO: Home care organization
IADL: Instrumental activities of daily living
MD: Medical doctor
NGT: Nominal group technique
QI: Quality indicator
RAI-HC: Resident Assessment Instrument-Home Care
U.S.: United States
WRHA: the Winnipeg Regional Health Authority
