# Supplementary material for: Home care quality indicators based on the Resident Assessment Instrument-Home Care (RAI-HC): a systematic review
Source: BMC Health Serv Res. 2020 Apr 29;20:366. doi: 10.1186/s12913-020-05238-x (PMC7191714; doi:10.1186/s12913-020-05238-x)
Supplement: Supplementary file 1 — Additional file 1. Search strategy used in PubMed. [file 12913_2020_5238_MOESM1_ESM.docx]

**Additional file 1.** Search strategy used in PubMed

("Health Status Indicators"[Mesh] OR "Quality Assurance, Health Care"[Mesh] OR "Quality Improvement"[Mesh] OR "Outcome Assessment (Health Care)"[Mesh] OR "Quality Indicators, Health Care"[Mesh] OR "Quality of Health Care/Nursing"[Mesh] OR client outcome*[tiab] OR patient outcome*[tiab] OR QI*[tiab] OR health status indicator*[tiab] OR "outcome-based quality improvement"[tiab] OR quality assessment*[tiab] OR quality indicator*[tiab] OR quality measure*[tiab] OR "care quality"[tiab] OR "quality of care"[tiab] OR "quality of health care"[tiab] OR "quality of healthcare"[tiab] OR "quality of nursing care"[tiab]) AND

(InterRai[tiab] OR inter-Rai[tiab] OR mds-hc[tiab] OR "minimal data set"[tiab] OR "minimum data set-home care"[tiab] OR Rai-HC[tiab] OR resident assessment instrument*[tiab])

Note: the [tiab] field qualifier in PubMed search the following fields: title, abstract. * is a truncation symbol.
